# Neutrophils, as “Trojan horses”, participate in the delivery of therapeutical PLGA nanoparticles into a tumor based on the chemotactic effect

**DOI:** 10.1080/10717544.2019.1701141

**Published:** 2019-12-09

**Authors:** Jifu Hao, Junlan Chen, Meixiang Wang, Jing Zhao, Jianze Wang, Xingrong Wang, Yuhong Li, Hua Tang

**Affiliations:** a College of Pharmacy, Shandong First Medical University & Shandong Academy of Medical Science, Taian, PR China;; b The State Key Laboratory Breeding Base of Basic Science of Stomatology (Hubei-MOST) & Key Laboratory of Oral Biomedicine of Ministry of Education (KLOBM), School & Hospital of Stomatology, Wuhan University, Wuhan, PR China;; c Institute of Immunology, Shandong First Medical University & Shandong Academy of Medical Science, Taian, PR China;; d Shanghai Public Health Clinical Center, Fudan University, Shanghai, PR China

**Keywords:** Neutrophils, chemokines, Trojan horse, PLGA nanoparticles, paclitaxel

## Abstract

Inspired by the fact that leukocytes have innate phagocytic functions and oriented migration capabilities in response to chemoattractants, we have unveiled that endogenous neutrophils as “Trojan horses”, participate in the delivery of nanoparticles in an “i*n vivo* self-armed assembly” manner. Neutrophils were the main population to preferentially sequester the intravenous administrated nanoparticles with an average size of 260 nm. The pre-implantation of CXCL1-laden hydrogels could trigger and induce a targeted signal to attract an influx of neutrophils carrying the therapeutic goods to the desired position. In mouse models of melanoma, the combinatorial regimen of using the PLGA nanoparticles with the CXCL1 hydrogels exhibited superior tumor inhibition capability. This work leveraged the natural phagocytosis of neutrophile and the chemotactic effect of chemokines for targeted delivery. We believe this strategy will improve the therapeutic efficiency of nanoparticle-based delivery systems, especially when the chemokines are implanted at sites of surgical tumor removal, during cancer treatment at the clinic.

## Introduction

The effective delivery of active pharmaceutical ingredients (API) to specific disease sites, instead of other normal tissues/organs, remains a challenge, especially for tumor treatment (Jiang et al., 2017). The development of nanoparticles drug delivery systems (DDS), intended to surmount the nonspecificity of conventional chemotherapeutic agents, has gained great promise for better therapeutic efficacy and reduced side effects. The main design principle for nanoparticles-based DDS usually depends on the enhanced permeability and retention (EPR) effect associated with incomplete tumor blood vessels, which allows for nanoparticles to passively to leak and permeate into tumors (Choi et al., 2007). However, the classical EPR theory was doubted and a literature indicated that the EPR effect worked only in rodents rather than in humans, because of the heterogeneous tumor types and species differences (Danhier, 2016). In additional, the anatomical structure of the neoplasm showed that larger regions of necrosis in the centers of solid tumors, due to persistent hypoxia and rapid proliferation of malignant cells destroying blood flow, render therapeutic nanoparticles inaccessible to these regions. Another alternative approach is the modification of nanoparticulate surfaces with particular ligands or antibodies to target malignant tumor cells, called receptor-mediated active targeting (Maeda, 2013). The active targeting approach enables nanoparticles to aggregate at the tumor sites based on the number of receptors overexpressed on the cell surface and the selective affinity between the receptors and ligands. Systemically injected nanoparticles, regardless of the kind of formulation, inevitably encounter numerous physiological barriers en route to the disease site; upon entering the blood, these nanoparticles are firstly marked by opsonins, such as albumin, immunoglobulins and complements, via a process called opsonization, and are subsequently recognized and phagocytized by the mononuclear phagocyte system (MPS) (Parodi et al., 2013).

As for nanoparticulate-based targeted DDS, it is ideal to provide stealthy capabilities to prevent them from being sequestered by the MPS as they travel through systemic circulation. Only nanoparticles with prolonged circulating properties can offer the opportunity for tumouritropic disposition (Torchilin, 2011). Considered from this point, PEGylated nanoparticles were conventionally used because nanoparticles modified with PEG molecules can develop an orbicular hydrophilic corona outside the nanoparticle to prevent protein adsorption and consequently being captured by leukocytes. Unfortunately, the use of PEG cannot completely prohibit being cleared and, on the contrary, results in further activation of the human complement system (Parodi et al., 2013). From this perspective, nanoparticulate-based drug delivery systems have not yet reached their full therapeutic potential (Michor et al., 2011).

Nowadays, more studies have focused on an alternatively cell-derived biomimetic drug delivery strategy which encapsulated nanoparticles into a population of endogenous living cells by phagocytosis or endocytosis, including monocytes, macrophages, neutrophils, endritic cells and stem cells; or engineered with cell membranes and subcellular membranes coating (Chu et al., 2015, 2016; Eyileten et al., 2016; Vij et al., 2016; Kang et al., 2017; Huang et al., 2018; Cao et al., 2019). These results highlighted the advantages of cell membranes camouflage or using living cells as drug transporters due to their better biocompatibility, strong abilities to migrate across impermeable barriers and safeguard against external danger irritations. Generally, a series of isolated monocytes, neutrophils, macrophages or T cells derived from peripheral blood or tumor tissues were individually incubated with API and/or nanoparticles *in vitro* for a period to fabricate cellular carriers; then these loaded cells were infused back into the host for cancer therapy (Jijun et al., 2015; Xue et al., 2017). Although cell-derived biomimetic drug delivery strategies hold great benefits to cancer therapy, these developments are plagued by the potential for contamination in the process of cell isolation, culture and transfusion. Whether employing living cells or cell membranes, the possible impairment of the cellular bioactivity resulted from the time spent in cell purification and *in vitro* culture should be considered (Choi et al., 2012; Tan et al., 2015). Thus, it is necessary to find an alternative delivery strategy to address these limitations.

Considering the innate phagocytic function of leukocytes, the nanoparticulates present in the bloodstream cannot prevent being sequestrated by leukocytes. Here, we discussed whether these parts of endogenous leukocytes could be developed as innate “Trojan Horses” to deliver nanoparticles into regions where other approaches are inaccessible after they spontaneously capture the nanoparticles *in vivo*. Chemokines, a type of signal protein secreted by tumor or immune cells, are chemotactic cytokines used to regulate leukocytes movement. One typical response of leukocytes to chemokines is the rapid mobilization of immune cells to aggregate against the chemokine concentration gradient (Song et al., 2015). Therefore, we further asked if chemokines are implanted into tumor in advance, could these chemokines be exploited as trigger signals to direct immune cells toward the chemotactic source and induce more leukocytes carrying the therapeutic nanoparticles into the tumor? Subsequently, after arrival at the destination, these leukocytes discharge their packages to elicit cytotoxicity and inhibit tumor proliferation.

In this study, PLGA nanoparticles with different diameters were tailored to encapsulate paclitaxel (PTX) for antitumor therapy and the fluorescent probe DiD for imaging. Then, the fate of the nanoparticles in blood was assessed by flow cytometry to probe which type of leukocytes were responsible for capture of the nanoparticles with different sizes; we also investigated the viability of neutrophils especially after uptake of paclitaxel nanoparticles *in vivo*, to elucidate whether nanoparticle-loaded neutrophils were able to survive and deliver their cargoes to the tumors. After subcutaneous implantation of the PLGA-PEG-PLGA thermosensitive hydrogels laden with CXCL1 chemokines, the *in vitro* release of chemokines and the recruitment effect were investigated. Furthermore, we performed an antitumor evaluation in melanoma bearing mice to probe the feasibility of using neutrophils as trojan horses and chemotaxis for tumor treatment. We believe these studies have significant implications for nanoparticle based delivery systems.

## 2. Materials and methods

### 2.1. Materials and animals

The copolymers poly (lactic-co-glycolicacid) (PLGA, 0.64 dL/g) carboxyl block and poly (lactic-co-glycolicacid)-polyethyleneglycol-poly (lactic-co-glycolicacid) (PLGA-PEG-PLGA) were purchased from Daigang Biotechnology Co., Ltd. (Jinan, China). Paclitaxel (PTX) was supplied by Ciyuan Biotechnology Co., Ltd. (Shanxi, China). 1,1'-Dioctadecyl-3,3,3',3'-tetra methylindo- dicarbocyanine, 4-chlorobe nzenesulfonate salt (DiD) was obtained from Sigma-Aldrich (USA). Double-distilled water was used in all experiments and other reagents were of analytical grade.

C57BL/6J mice and BALB/c nude mice were provided by Shandong First Medical University Animal Center (Taian, China). All animal studies were conducted according to the Principles of Laboratory Animal Care, and the protocols were approved by Shandong First Medical University Animal Ethical Committee.

### 2.2. Fabrication of PLGA nanoparticles

To elucidate the relationship of tailored PLGA nanoparticles particulate size associated with the cellular uptake, three kinds of PLGA nanoparticles with well-defined particle sizes were fabricated as previously described (Hu et al., 2011; Choi et al., 2014). The fluorescent probe, DiD, encapsulated into the PLGA nanoparticles, was used to indicate the behavior of the nanoparticles *in vivo* when taken up by the different subsets of leukocytes. PLGA nanoparticles with relatively small diameters were produced by the nanoprecipitation method. Briefly, the polymer PLGA and fluorescent probe DiD were codissolved in acetone, then introduced dropwise into distilled water with constant stirring at 1000 rpm. After removal of the organic solvent and collection by centrifugation, the obtained nanoparticles were redispersed into distilled water and lyophilized for further use. In addition, to generate nanoparticles with anticipative medium diameters, an alternative approach was applied by means of a double emulsification/solvent evaporation technique. A portion of the PLGA/DiD in ethyl acetate (as oil phase) was emulsified with a 0.1% PVA solution (as inner water phase) to form the primary emulsion (W/O) by probe ultrasonication. The primary emulsion was then dispersed into an aqueous solution containing the stabilizer PVA to form the secondary emulsion (W/O/W). The same procedure was analogous to that used to produce even larger particle sizes, except for alteration the ultrasonic intensity and duration and the adjustment of the polymeric PLGA concentration in the oil phase. After completely removing organic solvent in the emulsion, the nanoparticles were obtained via centrifugation and washed three times to remove residuary PVA. All samples were freeze-dried for future use.

The physicochemical properties of the prepared nanoparticles, such as their size and size distribution (polydispersion index, PDI) by laser light scattering, surface morphology by TEM, were also measured.

### 2.3. Analysis of PLGA-NPs uptake in blood by flow cytometry

To determine which types of leucocytes populations would selectively sequester the nanoparticles, a detailed analysis of the DiD-PLGA nanoparticles distribution in bloodstream was performed in C57BL/6J mice after intravenous administration. Mouse blood was drawn by retroorbital puncture with heparinized tubes at 1 h, 3 h and 6 h postintravenous injection of the same dose of DiD-PLGA nanoparticles with different particle sizes. For blood leukocyte cell surface staining, lysis of the red blood cells was performed with ammonium-chloride-potassium (ACK) lysing buffer (Gibco Life Technologies). The remaining immune cells were collected by centrifugation and resuspended in custom RPMI-1640 medium at 25 × 10^6^ cells/mL.

Then, blood leukocytes were incubated with a 2 μg/mL anti-Fcγ RIII/II receptor mAb (2.4 G2) (eBioscience) to block the Fcγ RIII/II receptors at 4 °C. After being washed with PBS, the cells were incubated for 15 min in PBS with cell-surface antibodies, then washed with PBS again and incubated for 15 min in PBS with Live cell/Dead cell Discrimination. Subsequently, the stained cells were analyzed on an LSRII (BD Biosciences), and the flow cytometry data was performed with Flow Jo software (Tree Star Inc.). The following antibodies were used in the experiments and purchased from eBioscience: anti-Ly6C (clone HK1.4), anti-Ly6G (clone 1A8).

### 2.4. Evaluation of neutrophils apoptosis after uptake PTX PLGA nanoparticles

As PTX is an extremely cytotoxic drug, it is necessary to demonstrate the drug-loaded PLGA nanoparticles should not affect the functions of neutrophils. The maintenance of normal function of neutrophils was a critical prerequisite to accomplish delivery of their chemotherapeutic cargo to the tumors. Therefore, we performed a time course of neutrophils apoptosis in the mice treated with PTX-PLGA nanoparticles with diameters of 260 nm. The experimental protocol was similar to the analysis of PLGA-NPs uptake in blood. After gating the sort of the DiD + and Ly6G + dual positive subsets, the Annexin-V FITC/PI kit was used according to the manufacturer's instructions. Stained cells were then analyzed by flow cytometry.

### 2.5. Preparation of CXCL1 chemokine laden PLGA-PEG-PLGA thermosensitive hydrogel

#### 2.5.1. Determination of phase transition temperature and gelatin time

The concentration of PLGA-PEG-PLGA affects the formation of the thermosensitive hydrogel. When a series of clear polymers solutions with concentrations ranging from 10% to 20% (w/v) were prepared, the phase transition temperature from the solution to the gel of the PLGA-PEG-PLGA copolymer was evaluated by the conventional vial inversion method (Ma et al., 2014). Approximately 2 mL of the obtained solution was introduced into the capped glass vial. Subsequently, the vial was immersed into water bath and heated from 10 °C to 50 °C with gradually increasing temperature. At each 1 °C interval, the tubes were inverted to monitor the flow status of the solution. After 10 min of incubation, the gel state was defined according to the criterion that no flowability was observed within 30 s in the inverted vial.

The gelatin time of the PLGA-PEG-PLGA solutions at different concentrations were also determined. The PLGA-PEG-PLGA aqueous solution stored in a glass bottle in 4 °C refrigerator was immersed into a water-bath at a constant 37 °C, and then the duration was recorded from the onset of the measurement until gel formation by the vial inverting method.

#### 2.5.2. *In vitro* CXCL1 chemokine release

The CXCL1 chemokine was dispersed into 15% (w/v) PLGA-PEG-PLGA copolymer aqueous solution to form a homogeneous clear solution at room temperature. The *in vitro* release test was performed by the membrane-less erosion experiments. Briefly, 2 mL CXCL1 loading copolymer solutions were introduced into a glass vial and incubated at 37 °C for 10 min to form the gel, then 2 mL of PBS (pH 7.4) was slowly added to the top of the formed gels as the release medium. Subsequently, the vial was placed into the oscillation apparatus at 37 °C with reciprocating vibrating frequency at 75 rpm.

At predetermined time intervals, the upper release medium was withdrawn, and the remaining gels were accurately weighed. Then, an equal amount of fresh PBS solution was added to maintain the original volume. The weight change of the gel was recorded to depict the degradation profile. The content of released CXCL1 in the medium was determined according to BCA measurement. Each data point was performed in triplicate.

### 2.6. *In vivo* imaging system (IVIS) evaluation of the recruitment effect

PLGA nanoparticles containing the near-IR fluorescent probe DiD were used for IVIS fluorescence detection. To assess whether the chemokines enabled neutrophils packaged with fluorescent DiD-PLGA nanoparticles to raise at the sites where the CXCL1 implanted, *in vivo* imaging system was used to evaluate the recruitment effect. After subcutaneous injection of the PLGA-PEG-PLGA thermosensitive hydrogel laden with or without CXCL1, the 6-week old BALB/c nude mice were intravenously administered PLGA nanoparticles into tail vein with an equivalent DiD content. Mice were then imaged by IVIS (Caliper, Hopkinton, MA) (DID filter: *λ*ex = 620 nm; *λ*em =650 nm) at predetermined time intervals of 0.5 h, 1 h, 2 h, 4 h, 6 h, 8 h, 12 h and 24 h postinjection under anesthesia. Fluorescence images of each sample were captured using Living Image^®^ 3.1 software.

### 2.7. *In vivo* antitumour activity

The *in vivo* tumor inhibition experiment by the use of chemokines to drive therapeutic-laden neutrophils for cancer therapy was conducted on 6-week-old female C57BL/6J mice with subcutaneous inoculation of 1 × 10^6^ syngeneic B16/F10 melanoma cells. As the tumor volume reached approximately ∼100 mm^3^, the tumor bearing mice were randomly assigned into four groups with eight mice in each group. Then, the tumor-bearing mice were subjected to treatment with: (1) PBS (the control group), (2) PTX liposomes, (3) PTX-PLGA NPs and (4) PTX PLGA NPs and CXCL1 (synergetic group of implantation of CXCL1 hydrogel in advance at the tumor sites and introvenously injection of PTX-PLGA). The mice received one injection in a 2 day interval and the treatments were repeated five times. The evaluation of antitumor efficacy and safety were measured by monitoring the tumor volume and body weight of the mice every 2 days during a period of 15 days. The tumor volume (V) was determined according to the following equations:

$$V=L\times W^{2}/2$$

where L and W represent the longest and shortest diameters (in mm) of the tumor mass, respectively (Wang et al., 2014). After the mice were sacrificed at the end of the experiment, the tumors were harvested and weighted. Then, the tumor inhibition rate was calculated. The tumor tissues were embeded into paraffin sections and hematoxylin and eosin (H&E) staining was carried out.

Furthermore, in order to evaluate the potential toxicity to the mice when treating with the different formulations of PTX during the therapeutic process, the samples of the major organs including the heart, liver, kidney and lung, were collected for histological tissue analysis. The obtained tissue samples were also performed H&E staining and the images were captured by Caseviewer 2.0 (pannoramic 250/MIDI,3DHISTECH, Hungary).

## 3. Results and discussion

### 3.1. Fabrication of size-dependent PLGA nanoparticles

**Figure 1.**
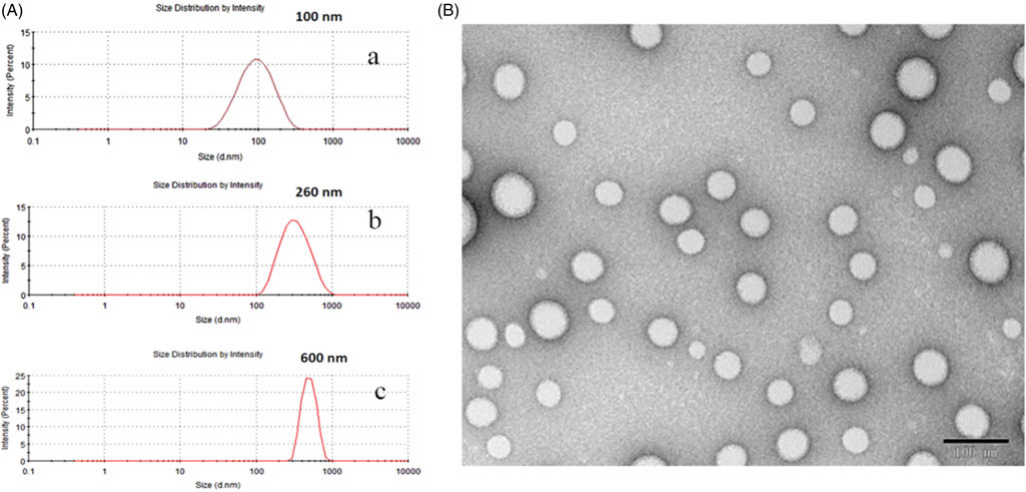
The particle size distribution (A) of about 100 nm diameters (represented small particle size) by nanoprecipitation method (a); of about 260 nm diameters (represented medium particle size) by an emulsification/solvent evaporation approach (b); of about 600 nm diameters (represented large particle size) by an emulsification/solvent evaporation approach (c); and TEM morphology (B) of PLGA nanoparticles.

Due to their better biodegradable and biocompatible properties, PLGA nanoparticles are broadly used for transporting active anti-tumor drugs and/or other substances inside their matrix shield (Vij et al., 2016). Two commonly used approaches, namely, the nanoprecipitation and emulsification solvent evaporation method, were used to generate size-dependent PLGA nanoparticles. As shown in Figure 1(A), the use of the nanoprecipitation method produced particle sizes below 100 nm with narrow size distributions (PDI < 0.3), while the double emulsification solvent evaporation method yielded PLGA nanoparticles with a diameter range of approximately 260 nm and 600 nm. In addition, TEM was employed to depict the morphology of the PLGA nanoparticles dispersed in the aqueous solution, as shown in Figure 1(B). From the TEM image, we found that the prepared PLGA nanoparticles presented a spherical structure with a smooth surface.

As for nanoprecipitation method, the developed PLGA nanoparticles with a mean particle size of 100 nm involved a spontaneous gradient-driven diffusion of amphiphilic organic solvents into a continuous aqueous phase. Rapid commutation of the organic solvents into miscible phase resulted in a decreased interfacial tension and the release of interfacial free energy between the two phases, which increased the surface area and led to polymer molecule precipitation on the interface to form nanodroplets after displacement of organic solvents that are miscible with water. After removing the organic solvents, the final nanoparticles were obtained via solidification and centrifugation (Danhier et al., 2012).

In the emulsification solvent evaporation method, the organic phase was primarily emulsified in the aqueous phase to form O/W emulsion droplets. Since this method produced particles by evaporating the organic polymer solution from the emulsified inner phase droplets (Birnbaum et al., 2000), the successful size control depended upon the manipulation of emulsion droplets. We found that the emulsion droplets correlated with both polymer concentration, ultrasonic intensity and emulsification process time. When the polymeric concentrations reached to 20 mg/mL, the ultrasonic intensity was limited to 200 W, and the ultrasonic duration was 2 min, the uniform sized nanoparticles with mean diameters of 600 nm were obtained. However, as the polymeric concentrations decreased to 10 mg/mL, the ultrasonic intensity increased to 400 W, and the ultrasonic duration extended to 6 min, and nanoparticles with mean diameters of approximately 260 nm were acquired. The imposed ultrasonic energy could break the emulsified droplets into small nanodroplets, which tends to produce relatively small particles at ultrasonic high intensity.

### 3.2. Neutrophils are responsible for capture PLGA-NPs in bloodstream

Due to the innate protective mechanisms of the body, systemically administered nanoparticles, upon entering the blood, are subjected to opsonization and then cleared by the MPS. Inspiration of this inherent phagocytic function of leukocytes, we hypothesized that some subsets of leucocytes may be exploited as transporters for meidation of nanoparticles delivery after uptake of the therapeutic nanoparticles *in vivo*. To better elucidation the distribution behavior of the PLGA nanoparticles with different particle sizes *in vivo* after intravenous administration, flow cytometry was exploited to define which types of leucocytes were involved in the uptake of PLGA nanoparticles in the bloodstream.

**Figure 2.**
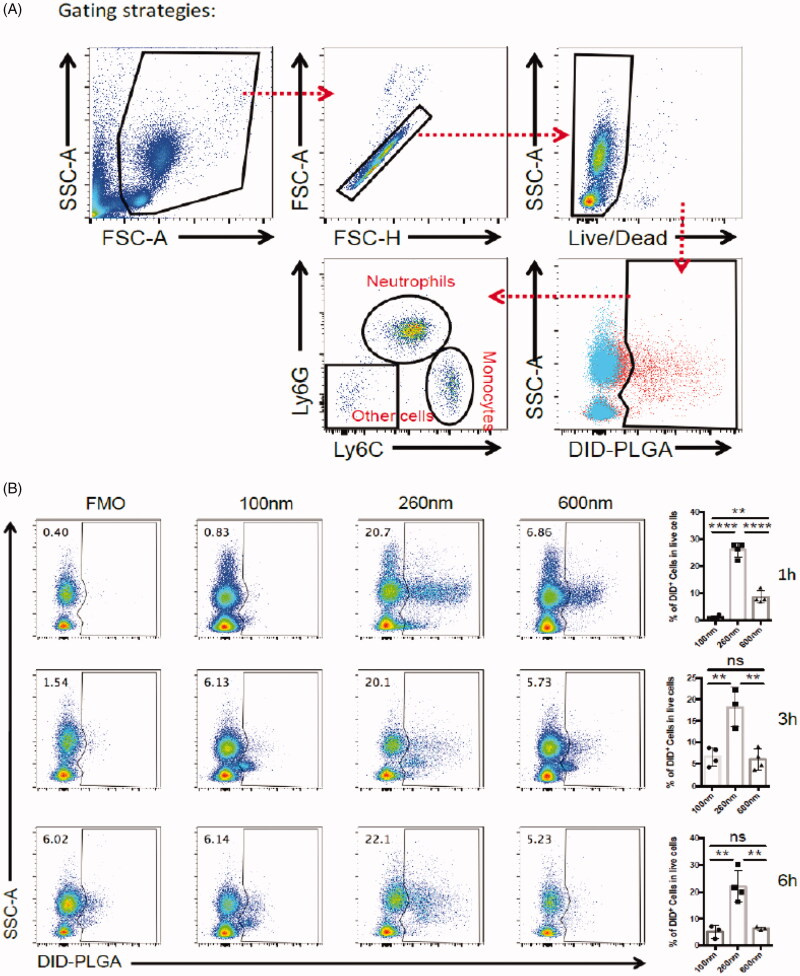
Flow cytometry analysis strategy of PLGA in circulating WBCs. (A) The gating strategy after injection of DID-PLGA nanoparticles and staining with specific antibodies for flow cytometry analysis. (B) The amount of different of sizes of DiD-PLGA nanoparticles internalized into the leukocytes at various times.

The gating strategies for flow cytometry analysis are shown in Figure 2(A). First, we sorted the living cellular population with positive DiD fluorescence signals to represent the phagocytic effect and to evaluate which nanoparticles particle sizes were prone to internalization into the corresponding cells after cellular staining. Then, the living cells with positive DiD fluorescence were further identified and sorted into cell subpopulations including neutrophils, monocytes and other cells. As depicted in Figure 2(B), we found that neutrophils, not monocytes, were the main subsets to preferentially internalize the nanoparticles of 260 nm diameters. This result means that neutrophils participated in the phagocytosis of PLGA nanoparticles in blood circulations. We can not find a reasonable explanation to understand why neutrophils preferentially sequestrated PLGA nanoparticles of 260 nm diameters; neutrophils in mice only account for approximately 2–30% of all leukocytes, although lymphocytes are dominated in the innate immune system.

In circulation, neutrophils patrol tissues and perform surveillance of danger signals to trigger immune defenses (Nicolás-Ávila et al., 2017). Due to their intrinsic phagocytic capabilities, after uptake of the foreign nanoparticles, neutrophils become transporters to deliver therapeutic nanoparticles. Therefore, each cell can potentially serve as a “Trojan Horse” to evade the immune system and deliver therapeutics into inaccessible tumor regions. We proposed this strategy as an “*in vivo* self-armed assembly” manner, which revealed that efficient delivery nanoparticles not only depend on their free forms without binding to the plasma proteins, but also involve in the participation of neutrophils in nanoparticles delivery. These results disclose that neutrophils play an important role in drug delivery that had not been identified in conventional nanoparticle targeting delivery systems.

Apart from the abovementioned in situ “*in vivo* self-armed assembly” method, another approach to fabricate a neutrophils-mediated delivery system was to incubate nanoparticles with neutrophils *in vitro*, which we suggested as an “*in vitro* assembly” method. Neutrophils captured nanoparticles via spontaneous endocytosis or phagocytosis pathways during the incubation process *in vitro*; subsequently, neutrophils carrying nanoparticles were reinfused to examine whether these neutrophils could deliver the nanoparticles to disease sites (Xue et al., 2017). However, this direct application of neutrophils as transporters for drug delivery was limited because the neutrophils are terminally differentiated cells with a half-life of only 7 h, and the potential hazard of a cytokine storm arises from the introduction of a large number of extra neutrophils (Coffelt et al., 2016; Dong et al., 2017; Chu et al., 2018).

### 3.3. Uptake of PTX PLGA nanoparticles does not impact neutrophils viability

**Figure 3.**
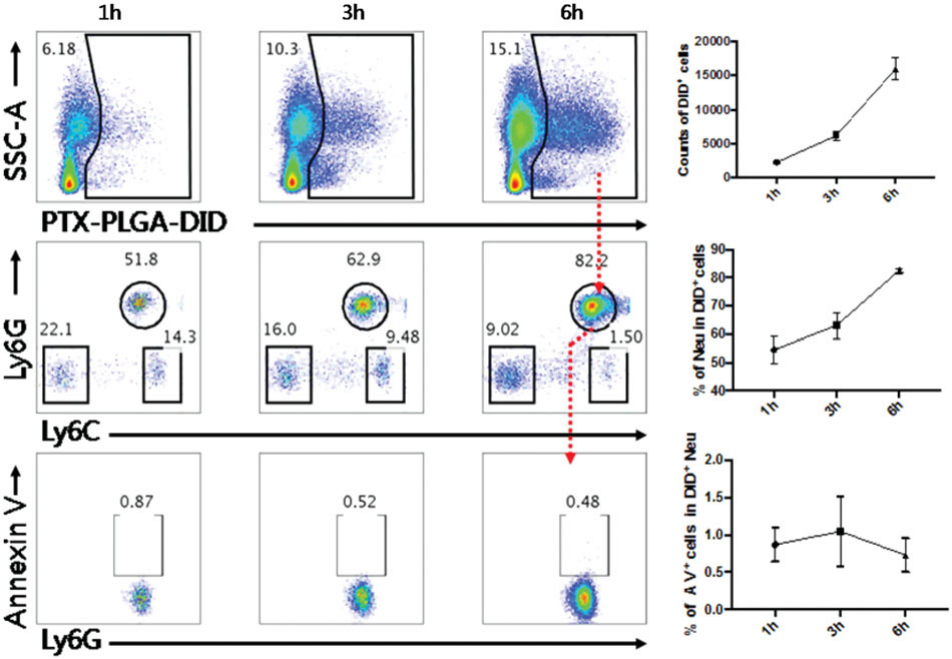
Flow cytometry analysis of the percentage of apoptotic neutrophils after PTX-PLGA nanoparticle uptake.

As neutrophils mediated drug delivery, the normal physiological function of these cells should not be impaired after nanoparticles are taken up by living cells *in vivo*. Therefore, the effect of drug loaded nanoparticles on cellular viability should be considered. To assess whether the nanoparticle encapsulated into the neutrophils impacted the viability of these vehicle cells *in vivo*, the Annexin V/PI dual labeling approach was performed to probe the degree of neureophil apoptosis after loading with the PTX-PLGA nanoparticles. As shown in Figure 3, the proportion of circulating neutrophils taking up nanoparticles accounted for 51.8%, 62.9% and 82.2% within 1, 3, 6 h, respectively, which implied that neutrophils were the predominant cells responsible for the sequestration of nanoparticles. The apoptotic cells accounted for ∼0.87%, 0.52%, 0.48% at the time points of 1, 3, 6 h, respectively. The results showed that the PTX PLGA nanoparticles do negligible harm to neutrophils, even after long exposure of neutrophils to the PTX-PLGA nanoparticles *in vivo*. These outcomes suggested that fabrication of PTX into PLGA nanoparticles provided sufficient safeguard against the impairment of neutrophils physiological functions. Hence, neutrophils would be potentially regarded as useful transporters to deliver therapeutical nanoparticles *in vivo*.

### 3.4. Thermogelling properties of gel and *in vitro* release studies

We used biocompatible thermosensitive hydrogels of PLGA-PEG-PLGA for the delivery of the CXCL1 chemokines due to their special “*in situ*” features. We hypothesized that these thermosensitive hydrogel systems injected *in vivo* could maintain sustained release of the CXCL1 chemokines. Therefore, in this section, CXCL1 chemokine-laden polymer hydrogels were prepared and their properties were discussed.

**Figure 4.**
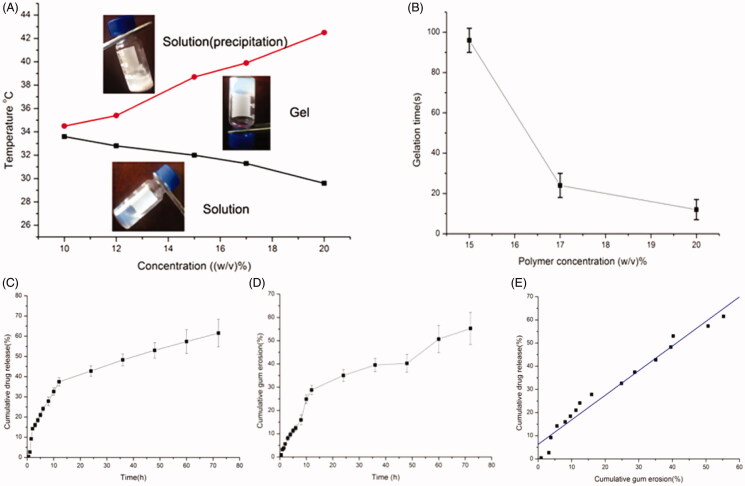
(A) Sol − gel phase transition diagram of the PLGA − PEG − PLGA aqueous solutions with increasing temperature. (B) Gelation time of the PLGA − PEG − PLGA aqueous solutions with various concentrations at 37 °C. (C) *In vitro* release of BSA from the polymer gels. (D) Profiles of the cumulative erosion of PLGA-PEG-PLGA hydrogel. (E) Profiles of the correlation between drug release and cumulative gel erosion.

Different amounts of polymer were completely dissolved in double distilled water at 4 °C to form a series of clear solutions with concentrations ranging from 10% (w/v) to 20% (w/v). As the temperature elevated from 20 °C to 50 °C, the solutions exhibited typical a “sol-gel” phase transition in the tube inverting experiments. The thermosensitive phase diagram was presented in Figure 4(A). As the temperature increased, a reversible sol-gel phase transition took place and a nonflowing semisolid transparent gel appeared as the concentration of sample was beyond the critical gelation concentration. The occurrence of gelation was mainly associated with the hydrogen bonding between the hydrophilic PEG segments of the copolymer chain and water molecules (Alexander et al., 2013).

As also shown in Figure 4(B), there was an obvious correlation between the sol-gel transition temperature and polymer concentration; the phase transition temperature declined with the increase of polymer concentration. This dependence of the sol to gel transition temperature on the polymer concentration was ascribed to higher polymer concentrations facilitating the formation of the copolymer network. However, further increase in the temperature caused additional phase precipitation, broke the hydrogen bond and further resulted in dehydration of the PEG shells.

Moreover, the duration of the gelation formation was surveyed at 37 °C to imitate the body temperature. It was found that the gelation time of the PLGA-PEG-PLGA aqueous solution shortened as polymer concentration increased, which greatly depended on the polymer concentration. When the polymer concentration varied from 15% to 20% (w/v), the gelation time declined from ∼96 s to 15 s, which suggested that the increase in polymer concentration facilitated gel formation.

Here, we found the gelation transformation temperature of the polymer started from 32.0 °C to 38.7 °C at the 15% concentration, which suggested that phase transition was within the scope of body temperature at this concentration. Additionally, the copolymer with 15% concentration did not present a rapid phase transition at room temperature. Consequently, a 15% concentration of the thermosensitive gel system was chosen for further use.

*In vitro* drug release and gel erosion studies were synchronously carried out. As shown in Figure 4(C,D), the release profiles of the model drug from the hydrogels at 37 °C exhibited a sustained release over 3 days. From the slope of the in vitro drug release curve, the first phase of the CXCL1 release from gels indicated a rapid release profile at the initial stage followed by an extended drug release profile. Furthermore, the gel erosion behavior showed a similar tendency. Subsequently, the correlation between the amount of drug released from the gel and the quantity of dissolved gel at the same time points was fitted. The profile of the fitted curve between drug release and cumulative gel erosion was depicted in Figure 4(E). We found that the release of drug was linear with the corrosion of the gel, which presented that the drug released slowly with the dissolution of the gel.

It is generally accepted that the rapid drug release observed at the initial stage involved in some drugs adsorbed on the gel surface. However, at the sustained release phase, bulk gel erosion predominantly induced the drug release from this system. When large molecular protein drugs were loaded, the intricate network of the hydrogel rendered them unable to escape from the gel freely. Other factors, such as the hydrogen bonds and static electric interaction between protein drugs and PLGA-PEG-PLGA molecules, made it more difficult for the protein molecules to diffuse out the gel.

The sustained erosion of the PLGA-PEG-PLGA gel was involved in both the cleavage of the polymeric chain through hydrolysis and the solubility of copolymers in the release medium. Considering the particular triblock structures of PLGA-PEG-PLGA, the higher the hydrophobic proportion of lactic acid was, the lower was the degradation rate.

The drug release data were fitted according to zero-order and Higuchi kinetic models, which were used to evaluate whether the release mechanism of the drug release follows bulk erosion or not. The large molecules did not diffuse freely through the small pores of the PLGA–PEG–PLGA hydrogels (zero or first order) or diffusion (Higuchi). *R* regression analysis of the mathematical model was used to judge the suitable release model (Cai et al., 2012). The *in vitro* data of the model drug release from the hydrogels was described by Higuchi equation due to the larger value of *R* (0.943). It was reported that the drug release from the hydrogels includes in two principal phases; at the initial stage, drug molecules diffuse from the hydrogels, then the drugs release via the slow erosion of the hydrogels matrix (Pratoomsoot et al., 2008; Luo et al., 2015).

### 3.5. Recruitment effect of CXCL1 loaded PLGA-PEG-PLGA thermosensitive hydrogel

Based on our previous results that neutrophils can spontaneously engulf nanoparticles *in vivo,* we further elucidated whether the nanoparticles-loaded-neutrophils can successfully transport nanoparticles to the desired sites and whether the CXCL1-laden hydrogels can cause the neutrophils accumulation from the bloodstream to the injected sites where the chemokines CXCL1 was implanted in advance.

**Figure 5.**
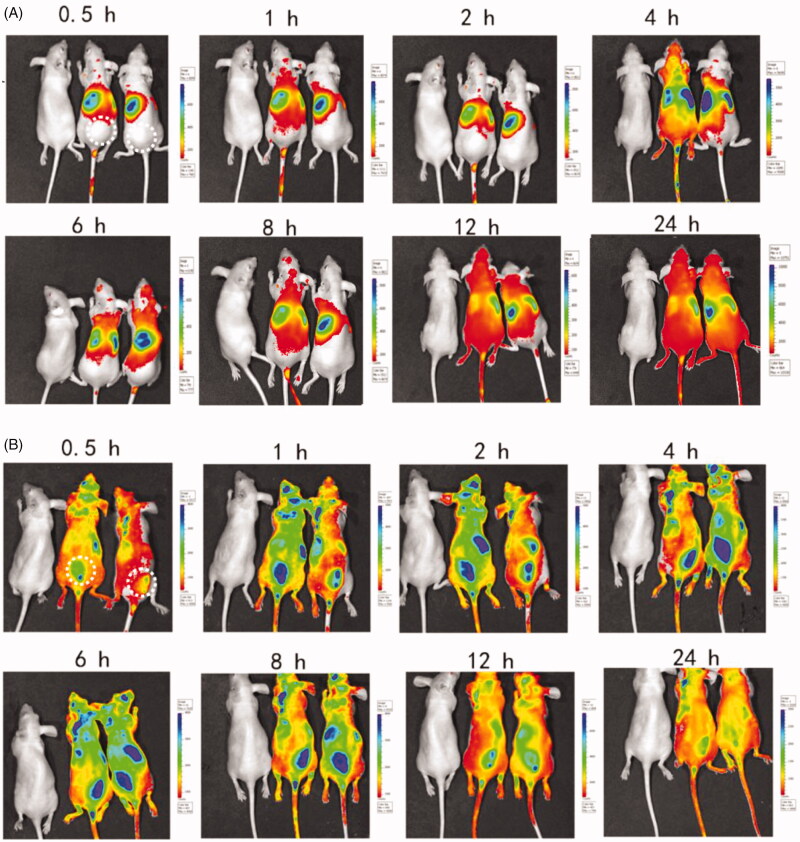
Biodistribution of DiD-PLGA nanoparticles after injected via tail vein of nude mice bearing B16/F10 melanoma cells. (A) *In vivo* near-IR fluorescence images of tumor-bearing mice treated without chemokines. (B) *In vivo* near-IR fluorescence images of tumor-bearing mice treated with chemokines. The dotted circle area of represents the injected site of thermosensitive hydrogels laden with chemokines.

After subcutaneous injection of the thermosensitive hydrogels with or without CXCL1 near the tumor sites, an image was captured at 0.5 h, 1 h, 2 h, 4 h, 6 h, 8 h, 12 h and 24 h after injection of the same dose of the DiD-PLGA nanoparticles via the tail veins. As depicted in Figure 5, no distinct DiD-fluorescent signals were observed at the injected site in the thermosensitive hydrogels without CXCL1 group; conversely, intensive fluorescent signals appeared in the group with CXCL1. Additionally, the fluorescence intensity of the CXCL1 group gradually increased from 1 to 8 h and the fluorescence signals at the administered site remained in a steady state from hours 4 to 8. These results demonstrated that that neutrophils participated in sequestrating the alien nanoparticles and delivering these fluorescent nanoparticles. Neutrophils are ready to respond to inflammatory stimuli and migrate from the circulation to infection sites via the initiation of inflammation (Kruger et al., 2015). Additionally, the sustained release of CXCL1 from the hydrogels developed concentration gradients of chemoattractants, which attracted more neutrophils with fluorescent cargoes to move against the concentration gradients of the chemokines and accumulate at the injected location (Xue et al., 2017). Hence, if the antitumor active ingredients were packaged into neutrophils, the oriented movement of the neutrophils could correspondingly deliver the cargoes to the desired sites with the assistance of chemokines. Therefore, the recruitment effect of CXCL1 hydrogels demonstrated the feasibility of using chemokines to increase an influx of neutrophils carrying active agents to the target sites.

### 3.6. *In vivo* therapeutic efficacy

According to the above evidence that innate neutrophils could serve as “Trojan horses” for potential nanoparticle delivery, and the “Trojan horses” could be devised to achieve oriented movement and penetration into inaccessible locations with the help of the implanted chemokines, we further validated the therapeutic efficiency via the combinatorial regimen against tumor growth. In this experiment, a B16/F10 murine melanoma model was established.

**Figure 6.**
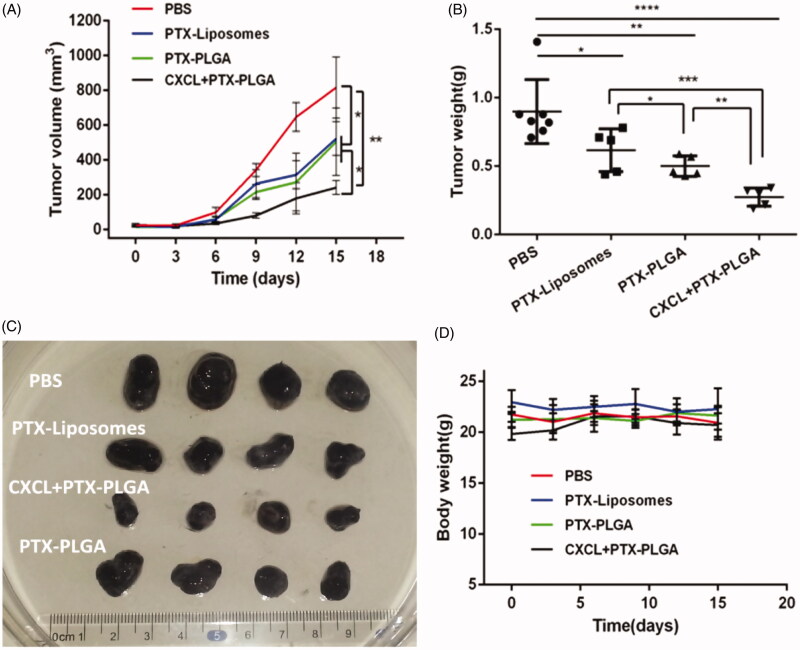
*In vivo* antitumor evaluation in B16 xenografted C57 mice. (A) Tumor volume over the treatment regimen (*n* = 8); (B) Tumor weight at the experimental endpoint; (C) Representative tumor tissues after treatment; (D) Change in body weight over the regimen.

As illustrated in Figure 6(A), tumor volumes in all PTX formulation treated mice were effectively decreased compared to those of the PBS-treated controls. In particular, it was notable that the cooperative application of PTX-PLGA and CXCL1 loaded hydrogels resulted in significant inhibition of tumor growth. Moreover, tumor inhibition ratios for the different treatments based on tumor weights, as shown in Figure 6(B,C), were in agreement with the results of the tumor volume experiment. Furthermore, combinatorial application of PTX-PLGA and CXCL1 loaded hydrogels showed the best tumor suppression efficiency (67.28%), followed by PTX-PLGA NPs (46.95%) and PTX-liposomes (31.44%). Additionally, we found that treatment with CXCL1 hydrogel alone could not effectively inhibit tumor growth (data not shown).

**Figure 7.**
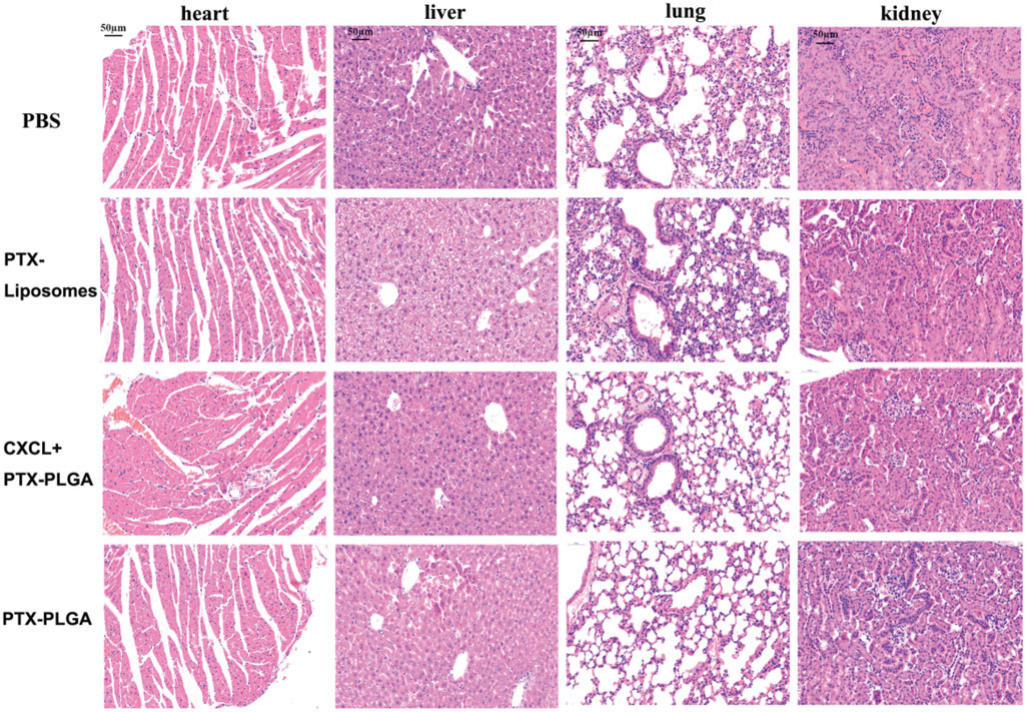
H&E staining images of major organs after treatment (heart, liver, lung, kidney. 40×, the scale bar represents 50 µm).

Additionally, it is generally accepted that body weight reduction is usually associated with drug toxicity after administration of an antitumor drug. Therefore, the alterations of body weights of the mice were monitored to evaluate the systemic toxicities of the treatment. As shown in Figure 6(D), there was no apparent loss in body weight during the experimental period for all the groups treated with the injection of PTX liposomes or PTX-PLGA nanoparticles, indicating low systemic toxicities of this treatment. Histological sections of the isolated organs were shown in Figure 7; the results indicated that there were no significant morphological changes in the H&E stained sections by microscopic examination, indicating that the vital organs were not seriously damaged in the treatment process.

**Figure 8.**
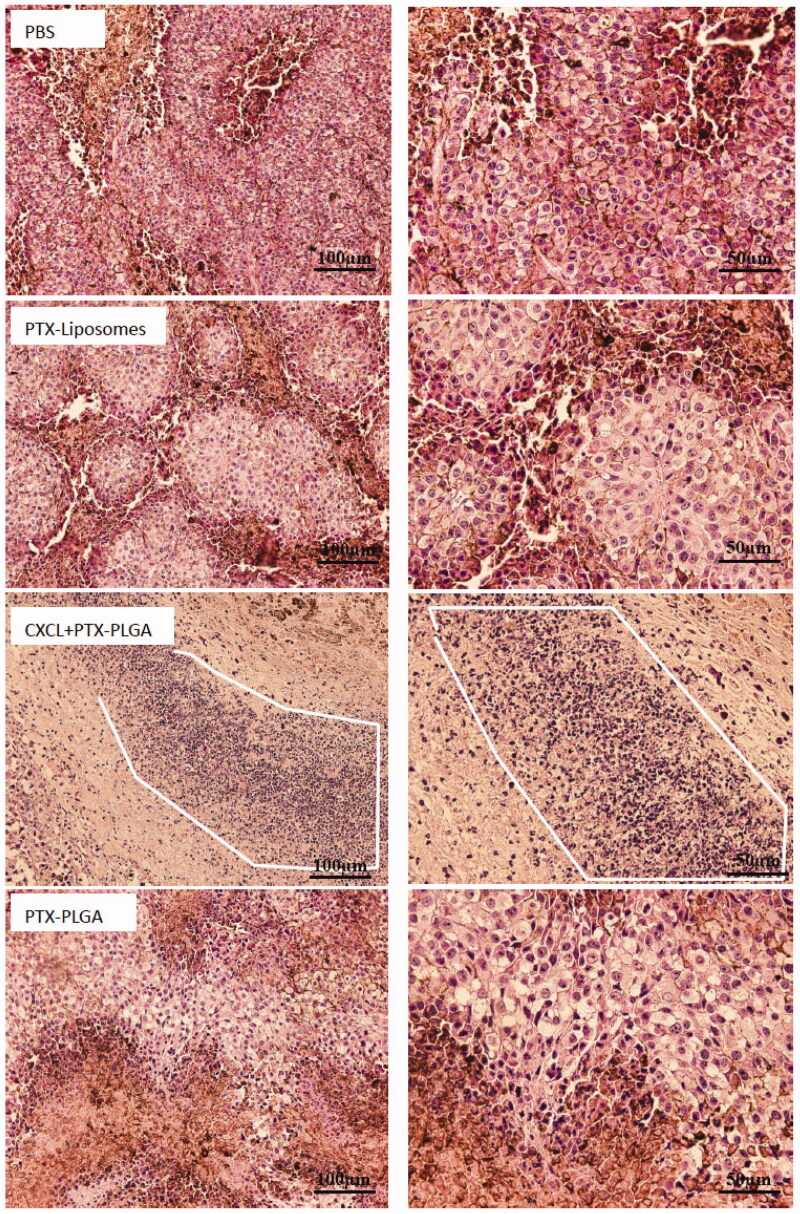
Histopathologic examination of tumor tissues after treatment with H&E staining. (left, 20×; right, 40×. The white line enclosed circle represents accumulation of neutrophils).

In Figure 8, the H&E pathological sections of tumors clearly illustrated that tumor cells with normal morphology were observed in the PBS group, while varying degrees of necrotic cells were presented in different PTX formulation groups. Moreover, the images distinctly showed that the nucleus grew smaller and the tumor cells lost their typical shapes in the group treated with the combination of PTX PLGA nanoparticles and CXCL1 hydrogels. Additionally, the group administered this treatment schedule exhibited the largest necrosis areas when compared with other groups, which demonstrated that the application of chemokines can better tumor inhibition efficiency. Moreover, we also found there were large amounts of neutrophils accumulation in the tumor sites. Therefore, neutrophils, serving as “Trojan horses” for the communication of therapeutic nanoparticles, and the influx of neutrophils, carrying therapeutic nanoparticles, resulted from the recruitment of chemokines present in the tumor location enabled more active ingredients into the target sites, which acted synergistically for tumor treatment.

According to the classical EPR effect theory, nanoparticles perform antitumor effect depending on the amount of nanotherapeutics that permeated into the tumor. Here we proposed that neutrophlis, as “Trojan horses”, participate in the delivery of therapeutical PLGA nanoparticles into a tumor based on the chemotactic effect. Bryan Ronain Smith advised that the Trojan horse mechanism in which the nanoparticles were selectively delivered to tumor via circulating blood cells was much more reliable for all the solid tumors and may be further applied in human disease for both diagnostic and therapeutic efficacy (Bryan Ronain et al., 2014).

According to the proposed “*in vivo* self-armed assembly” manner, nanoparticles delivery is not only dependent on the circulation leaking into tumor, which is a distinct difference from the classical EPR effect, but also is mainly related to the amount of the immune cells as potential carriers in the blood. Many published articles have demonstrated that neutrophils could be applied to deliver nanotherapeutics to inflammatory or tumor sites, which attributed to the rapid reaction of neutrophils to the inflammatory regions; the amount of neutrophils and even the number of neutrophiles increased to hundred-fold or more in a short period as a response to inflammation (Coffelt et al., 2016; Dong et al., 2017; Huang et al., 2018). Chemotaxis and tissue transmigration of neutrophils are critical for delivering drugs after nanotherapeutics are internalized by neutrophils (Chu et al., 2018). Subsequently, the targeted signals were adequately enlarged by the pre-implanted chemokines to recruit more nanoparticles-laden-neutrophils to the tumor sites. Additionally, the delivery of nanoparticles into the tumor via this targeting mechanism are associated with the amount of nanoparticles internalized into the neutrophils. Therefore, future interesting work will be required to rational design of nanoparticles and increased targeting specificity to neutrophiles intended to enhance the nanoparticles uptake in activated neutrophils.

## Conclusion

**Scheme 1.**
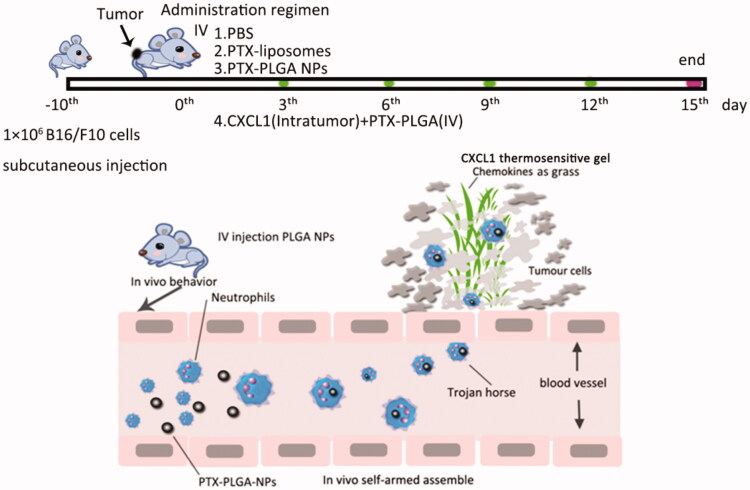
Schematic illustration of neutrophils as “Trojan horses” participation in the delivery of therapeutical PLGA nanoparticles into a tumor based on the chemotactic effect.

In summary, we have unveiled neutrophils participating in delivery nanoparticles in an “*in vivo* self-armed assembly” approach to achieve effective and accurate traffic, which includes both the use of innate neutrophils as “Trojan horses” to package therapeutic nanoparticles and the assistance of chemokines to initiate and amplify chemotactic signals to attract those Trojan horses to migrate into the site where the chemokines are implanted, schematic illustration has shown in Scheme 1. Therefore, we regard the chemokines as “grass” and believe that the Trojan horses can actively eat the “grass”. This work has significant implication for improvement cancer therapy by therapeutic nanoparticles, in particular, when the chemokines are implanted at sites of surgical tumor removal, during cancer treatment at the clinic. This work also highlights the double advantage of neutrophils phagocytosis and the recruitment via additional chemokines for targeted delivery. We believe this strategy will offer new opportunities to enhance the therapeutic efficacy of nanoparticle-based delivery systems, which potentially can be used for the treatment of diverse diseases.
